# Supplementary material for: Ligand Tuning of Localized Surface Plasmon Resonances in Antimony-Doped Tin Oxide Nanocrystals
Source: Nanomaterials (Basel). 2022 Oct 4;12(19):3469. doi: 10.3390/nano12193469 (PMC9565614; doi:10.3390/nano12193469)
Supplement: Supplementary file 1 [file nanomaterials-12-03469-s001.zip › Supporting Information with affiliations corrected.pdf]

nanomaterials

Supplementary Materials

Ligand Tuning of Localized Surface Plasmon Resonances in Antimony-Doped Tin Oxide Nanocrystals

Olexiy Balitskii [1,2,3,†], Oleksandr Mashkov [1,†], Anastasiia Barabash [4], Viktor Rehm [1], Hany A. Afify [1,5], Ning Li [4], Maria S. Hammer [4], Christoph J. Brabec [4,6], Andreas Eigen [7], Marcus Halik [7], Olesya Yarema [8], Maksym Yarema [8], Vanessa Wood [8], David Stifter [9] and Wolfgang Heiss [1,4,*]

[1] Institute-Materials for Electronics and Energy Technology (i-MEET), Department of Materials Science and Engineering, Friedrich-Alexander-Universität Erlangen-Nürnberg, Energy Campus Nürnberg, Fürtherstraße 250, 90429 Nürnberg, Germany
[2] Department of Electronics, Lviv Ivan Franko National University, Dragomanov Str., 50, 79005 Lviv, Ukraine
[3] Adolphe Merkle Institute, Fribourg University, 1700 Fribourg, Switzerland
[4] Institute-Materials for Electronics and Energy Technology (i-MEET), Department of Materials Science and Engineering, Friedrich-Alexander-Universität Erlangen-Nürnberg, Martensstraße 7, 91058 Erlangen, Germany
[5] Department of Laser Sciences and Interactions, National Institute of Laser Enhanced Sciences (NILES), Cairo University, Giza 12613, Egypt
[6] Helmholtz-Institut Erlangen-Nürnberg, Immerwahrstraße 2, 91058 Erlangen, Germany
[7] Organic Materials & Devices, Department of Material Science, Interdisciplinary Center for Nanostructured Films (IZNF), Friedrich-Alexander University Erlangen-Nürnberg (FAU), Cauerstrasse 3, 91058 Erlangen, Germany
[8] Institute for Electronics, Department of Information Technology and Electrical Engineering, ETH Zürich, 8092 Zürich, Switzerland
[9] Center for Surface and Nanoanalytics (ZONA), Johannes Kepler University Linz, 4040 Linz, Austria
* Correspondence: wolfgang.heiss@fau.de
† These authors contributed equally to this work.

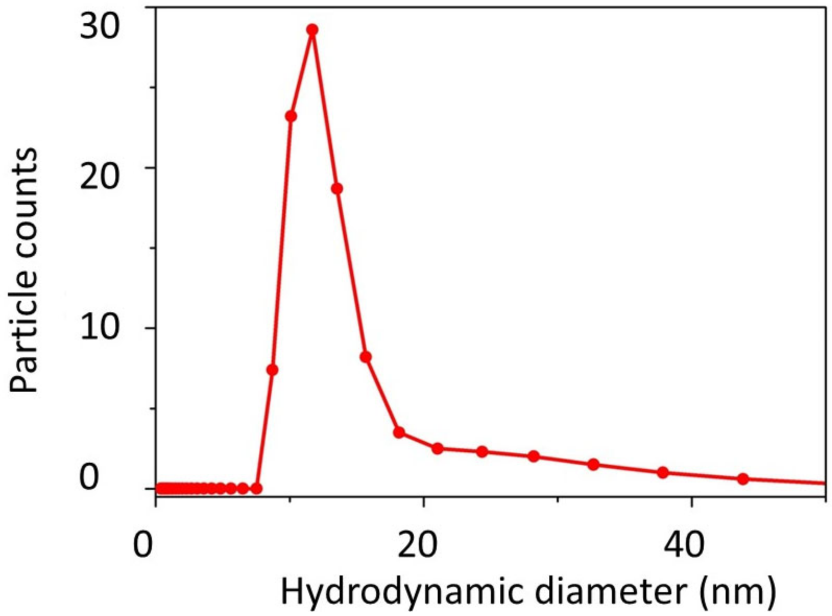

Figure S1. Hydrodynamic diameter. Size distribution of ATO NCs with 20 % Sb concentration and OA ligands as measured by dynamic light scattering.

Table S1. Composition. EDX data of ATO NCs with various Sb concentrations. For the measurements the NCs were deposited onto Si substrates. The data were averaged from at least five different areas for each film.

**ATO**	**20 %**	**10 %**	**5 %**	**0 %**
Element	at. %	at. %	at. %	at. %
O	70.2	70.4	71.8	71.0
Sn	24.6	26.5	26.3	29.0
Sb	5.2	3.1	1.9	N/A
Sn/Sb (%)	21.1	11.6	7.2	0

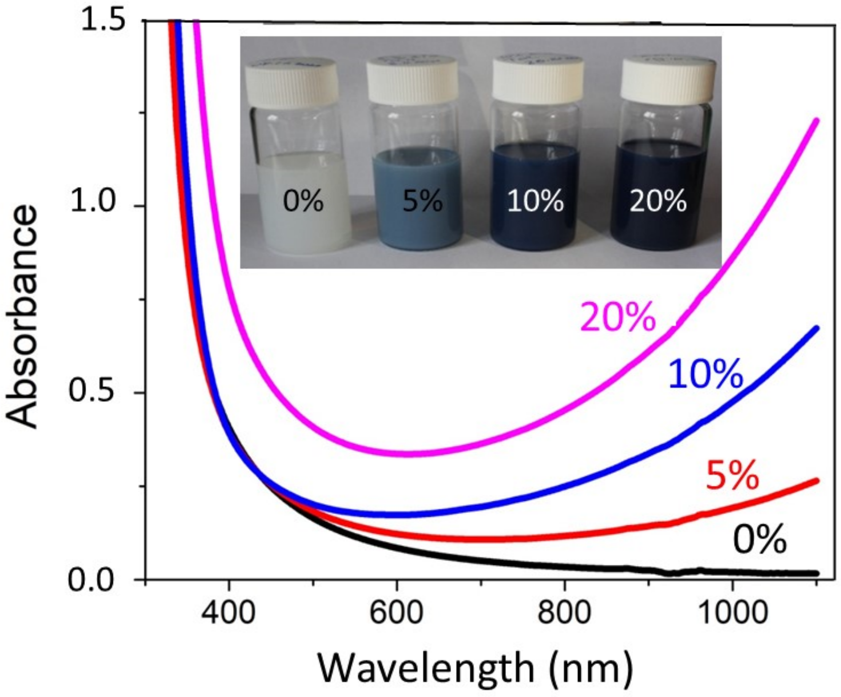

Figure S2. ATO in the visible. Photo (inset) and UV-vis-NIR spectra of OA-capped ATO NC solutions in toluene (concentrations: 100 mg/mL in the photo and 10 mg/mL for the absorbance spectra).

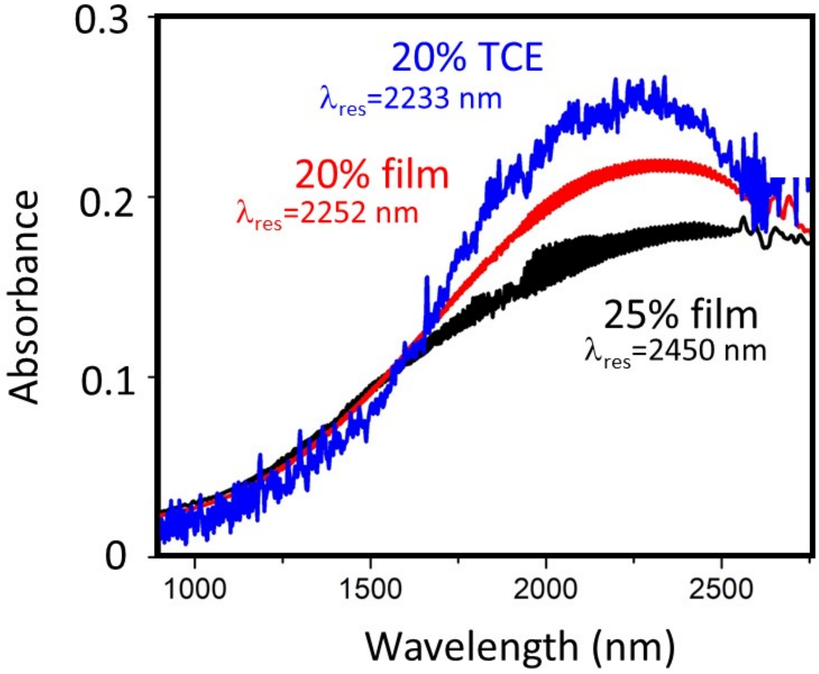

Figure S3. **From solution to film.** Absorbance of OA capped ATO NCs. Compared are NCs in solution to the NCs in film for 20% Sb concentration and NCs in film with 20 % and 25% Sb concentrations.

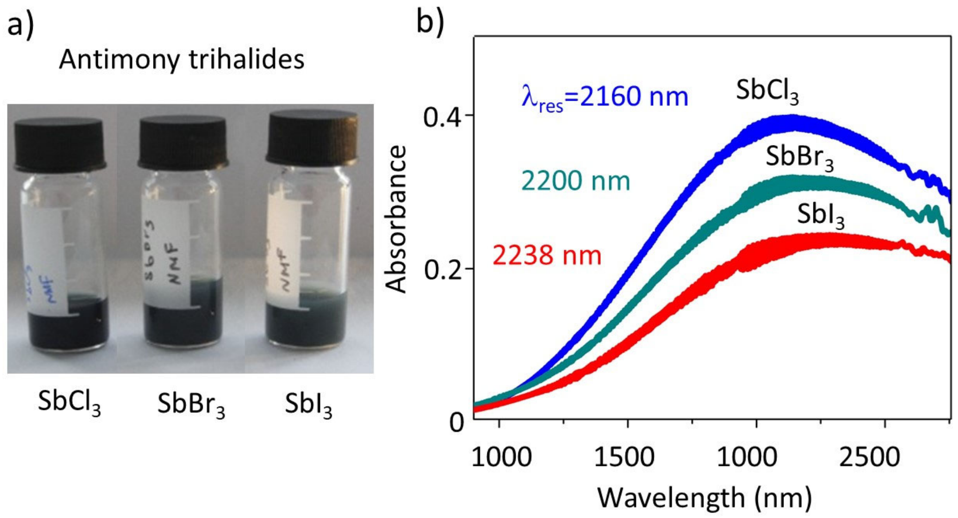

Figure S4. **Antimony-halide ligands.** ATO NCs with a Sb doping concentration of 20% after ligand exchange to Antimony trihalides. a) Photo of $SbCl_3$, $SbBr_3$ and SbI_3 covered NCs for which the absorbance spectra of the corresponding film samples are shown in b), indicating also the resonance wavelengths of the LSPR maxima.

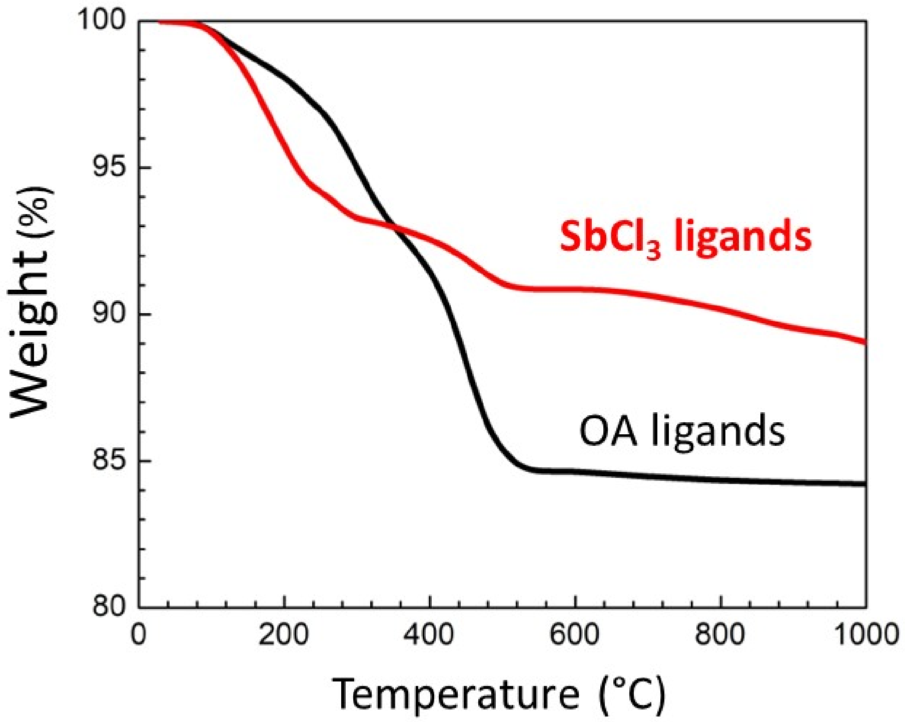

Figure S5. Ligand amounts. TGA from ATO NCs with 20 % Sb concentration, stabilized with long organic and short inorganic ligands.

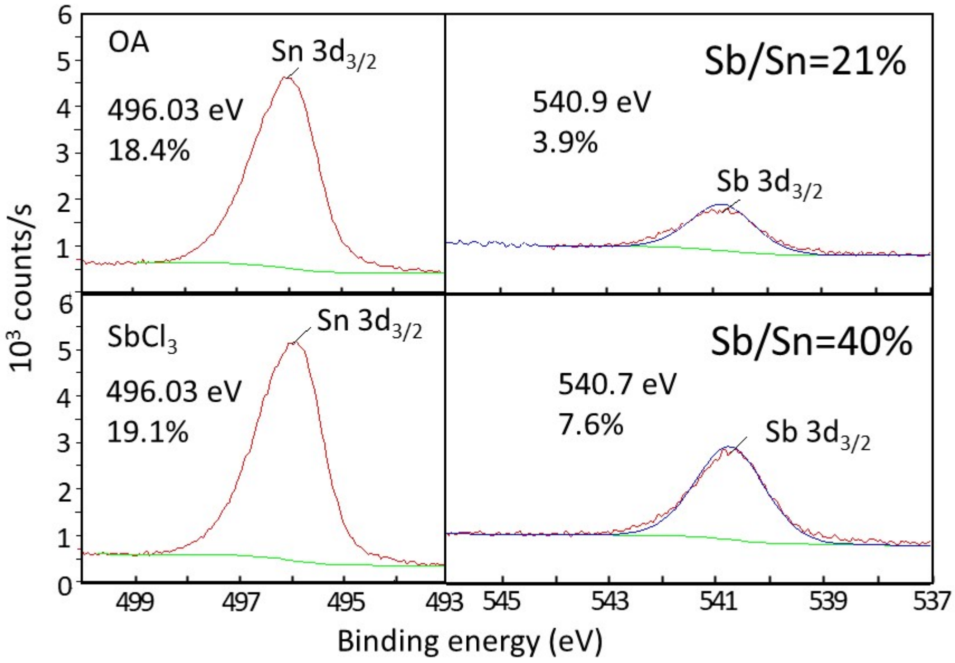

Figure S6. SbCl₃ ligand attachment. XPS spectra of ATO NCs with 20 % Sb content before and after ligand exchange for the Sn $3d_{3/2}$ energy region and the Sb $3d_{3/2}$ region.

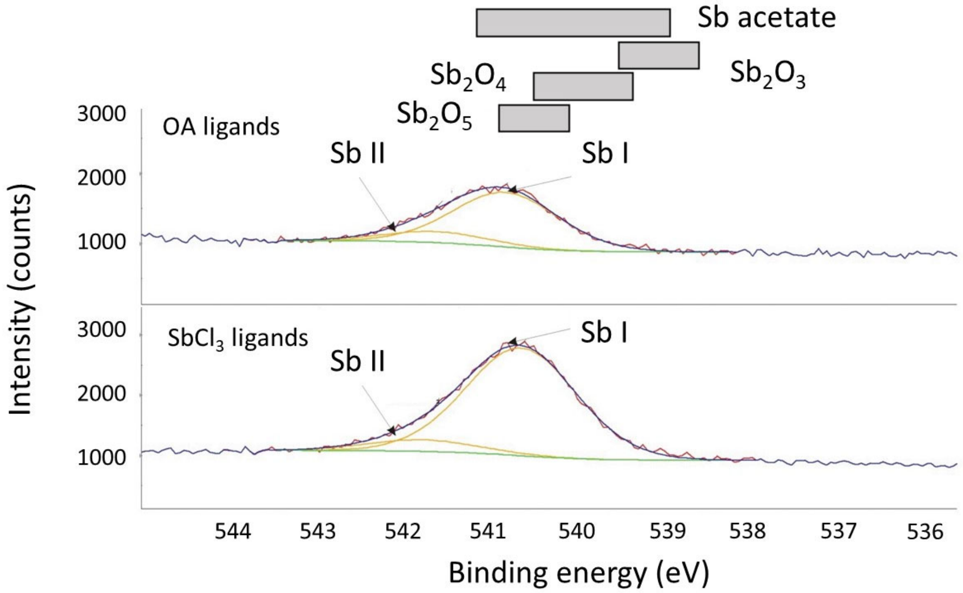

Figure S7. SbI and Sb II in XPS. XPS spectra of ATO NCs with 20 % Sb content before and after ligand exchange for the Sb $3d_{3/2}$ region including curve fits. Binding energy ranges of reference compounds are taken from Ref. 41 of the main text are shown as gray-colored boxes. From there also the separation of the peak in contributions called SbI and SbII are taken.
